# Epidemiological and Virological Characteristics of Influenza in the Western Pacific Region of the World Health Organization, 2006–2010

**DOI:** 10.1371/journal.pone.0037568

**Published:** 2012-05-29

**Authors:** Members of the Western Pacific Region Global Influenza Surveillance and Response System

**Affiliations:** Harvard School of Public Health, United States of America

## Abstract

**Background:**

Influenza causes yearly seasonal epidemics and periodic pandemics. Global systems have been established to monitor the evolution and impact of influenza viruses, yet regional analysis of surveillance findings has been limited. This study describes epidemiological and virological characteristics of influenza during 2006–2010 in the World Health Organization's Western Pacific Region.

**Methodology/Principal Findings:**

Influenza-like illness (ILI) and influenza virus data were obtained from the 14 countries with National Influenza Centres. Data were obtained directly from countries and from FluNet, the web-based tool of the Global Influenza Surveillance and Response System. National influenza surveillance and participation in the global system increased over the five years. Peaks in ILI reporting appeared to be coincident with the proportion of influenza positive specimens. Temporal patterns of ILI activity and the proportion of influenza positive specimens were clearly observed in temperate countries: Mongolia, Japan and the Republic of Korea in the northern hemisphere, and Australia, New Zealand, Fiji and New Caledonia (France) in the southern hemisphere. Two annual peaks in activity were observed in China from 2006 through the first quarter of 2009. A temporal pattern was less evident in tropical countries, where influenza activity was observed year-round. Influenza A viruses accounted for the majority of viruses reported between 2006 and 2009, but an equal proportion of influenza A and influenza B viruses was detected in 2010.

**Conclusions/Significance:**

Despite differences in surveillance methods and intensity, commonalities in ILI and influenza virus circulation patterns were identified. Patterns suggest that influenza circulation may be dependent on a multitude of factors including seasonality and population movement. Dominant strains in Southeast Asian countries were later detected in other countries. Thus, timely reporting and regional sharing of information about influenza may serve as an early warning, and may assist countries to anticipate the potential severity and burden associated with incoming strains.

## Introduction

Influenza, an acute viral infection characterised by fever with cough and/or sore throat, occurs as annual seasonal epidemics in winter or early spring in countries with temperate climates [1]. These yearly epidemics pose a substantial health burden arising from related complications such as lower respiratory tract infections and exacerbation of cardiopulmonary and other chronic diseases. Influenza burden in tropical or subtropical countries is not well-defined, although there is mounting evidence that prevalence and excess mortality from influenza are comparable to those seen in temperate countries [2], [3].

The Global Influenza Surveillance Network (GISN) was established by the World Health Organization (WHO) in 1952 and was re-named the Global Influenza Surveillance and Response System (GISRS) in 2011 [4]. The GISRS monitors the impact and evolution of influenza viruses and emergence of novel influenza viruses with pandemic potential. It also provides recommendations on suitable virus strains for inclusion in vaccines (twice yearly for northern and southern hemisphere seasons) and on diagnostic tests and antiviral drug sensitivity. The GISRS currently consists of six WHO Collaborating Centres (WHO CCs) for Reference and Research on Influenza (five for human influenza and one for animal influenza), four Essential Regulatory Laboratories (ERLs) and 136 WHO National Influenza Centres (NICs) in 106 WHO Member States.

**Figure 1:**
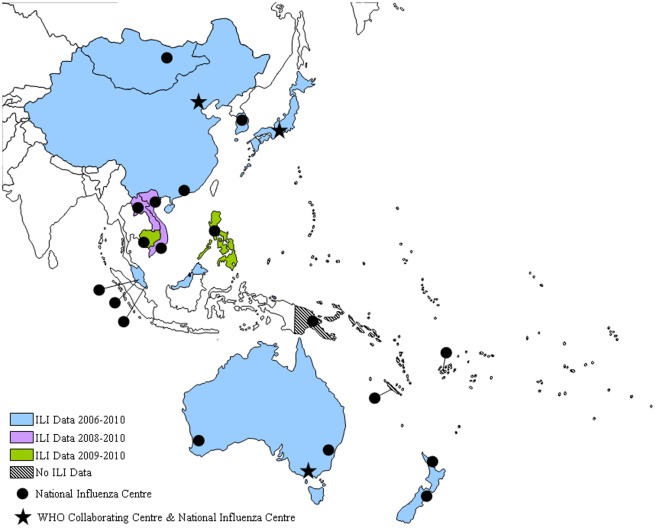
WHO's Western Pacific Region with National Influenza Centres and WHO Collaborating Centres on influenza, and years of ILI data contributed by each country/area.

The WHO's Western Pacific Region includes 37 countries and areas that span from the northern hemisphere through the tropics to the southern hemisphere (Figure 1). This region covers nearly one-quarter of the world's population with approximately 1.6 billion people. The GISRS in the region currently consists of 21 NICs in 15 countries, three WHO CCs, one each in Australia, Japan and China, and two ERLs, in Australia and Japan.

Progress that has been made in the development of influenza surveillance capacity in the Western Pacific Region was demonstrated by the rapid availability of data from 34 countries and areas during the influenza A(H1N1)pdm09 pandemic [5]. However, there has been limited reporting of epidemiological and virological data on seasonal influenza in the region [6]. This study describes the epidemiological and virological characteristics of influenza for 2006–2010 in the Western Pacific Region, with a view to supporting the development of future regional strategies and workplans to further strengthen influenza programmes.

## Methods

### Data Collection

Influenza-like illness (ILI) and influenza virus data for 2006–2010 were obtained from the 14 countries with National Influenza Centers (NICs) in the Western Pacific Region that participated in the Fifth Meeting of NICs in WHO's Western Pacific and South-East Asia Regions in June 2011. Participating countries included Australia, Cambodia, China (including Hong Kong), Fiji, New Caledonia (France), Japan, Lao People's Democratic Republic (PDR), Malaysia, Mongolia, New Zealand, the Philippines, the Republic of Korea, Singapore, and Viet Nam (Figure 1). Data were obtained from NICs and disease surveillance units with support of WHO country offices.

Data were collected using an email survey between April and October 2011. The survey included a questionnaire that ascertained qualitative data on ILI surveillance systems, case definitions applied and virological surveillance methods. The email survey also obtained data on number of weekly or monthly ILI cases reported and total number of consultations for any cause by age group. Age groups were categorized as 0 to <2 years, 2 to <5 years, 5 to <18 years, 18 to <65 years, and ≥65 years; however, alternative age groupings were accepted to allow for reporting differences by countries. These data were obtained using a reporting template developed in Microsoft Excel®.

Virological data were initially extracted from FluNet, the web-based data collection and reporting tool of the GISRS, to which NICs report the number of specimens processed for influenza virus testing and the number testing positive for influenza A(H1N1)pdm09, seasonal A(H1), A(H3), A(H5), A(not subtyped), B(Victoria), B(Yamagata) or B(lineage not determined). The extracted data were sent electronically to each participating country in March 2011 for confirmation. Using the above-mentioned Microsoft Excel® template, virological data by week were also requested from countries that had not reported to FluNet or had data missing in FluNet during 2006–2010. See Supplementary Table S1 for the type and period of data available from each country participating in this study.

### Data Analysis

ILI case definitions and surveillance systems were tabulated for each reporting country.

For data presentation and comparison, the raw data were tabulated and graphed, and countries were grouped according to geographic location and apparent similarities in ILI and virus circulation patterns. The groups are: (A) the Republic of Korea and Mongolia, (B) China, (C) Cambodia, Lao PDR, Malaysia, Philippines, Singapore and Viet Nam, and (D) Australia, Fiji, New Caledonia (France) and New Zealand. Percent ILI was calculated as a proportion of ILI cases per total consultations, and percent specimens positive for influenza was calculated as a proportion of influenza positive specimens per total specimens submitted for influenza testing. For each group of countries, the denominators (total consultations or total specimens tested) were first summed and then used to calculate regional proportions. Ratios for ILI cases and ratios of specimens positive for influenza could not be calculated for Japan, as denominators were not reported in the data collection template, thus it was not included in any of the groups. Case and specimen numbers were described in the results text.

## Results

Fourteen countries responded to the survey. ILI case definitions, surveillance approach and intensity varied across countries, as did the number of years of reporting.

The definition of ILI varied across sentinel surveillance schemes within countries and areas and across countries (Table 1). Seven countries and one area reported applying the WHO definition: a person with sudden onset of fever >38°C and cough or sore throat in the absence of other diagnoses. Other definitions applied by reporting countries used broader clinical symptoms, separated definitions for different age groups or limited the timeframe for onset of different symptoms.

**Table 1:** Surveillance and ILI case definitions in Western Pacific Region countries, 2006–2010.

Country	Surveillance System	ILI Case definition
Australia*	Approximately 250 general practitioner clinics	Fever (≥38°C), cough and fatigue
	65 Emergency departments	Emergency Departments: Fever (≥38°C) or feverishness plus at least one of the following respiratory symptoms: cough orsore throat
	Community online data collection	Cough and fever
Cambodia	8 hospitals	Sudden onset of fever of >38°C and cough orsore throat within 5 days
China	2006–2009: incremental increase from 197 to 556 sentinel hospitals and411 network laboratories	Sudden onset of fever of >38°C and cough or sore throat
	2010: 556 sentinel hospitals and 411 network laboratories	As above
Hong Kong (China)	Approximately 114 public and private outpatient clinics	WHO definition∧
Fiji	January-June 2009: 7 sentinel hospitals	WHO definition∧
	July 2009–December 2010: 13 sentinel hospitals	As above
Japan	3,000 pediatric and 2,000 internal medicine sites	Sudden onset of fever of >38°C, Upper respiratory infection and feeling tired.
Lao PDR	2007–2008: 3 hospitals	WHO definition∧
	2009–2010: 8 hospitals	As above
Malaysia	All government health clinics (approximately 600)	WHO definition∧
Mongolia	2006–2009: incremental increase from 30 hospitals and health centres to37 hospitals and 121 health centres	WHO definition∧
	2010: 37 hospitals and 121 health centres	As above
New Caledonia (France)	2 hospitals and 7 health centres	Sudden onset of fever ≥38°C (or shiver if temperature not available) and cough (or sore throat)
New Zealand	2006–2008, 2010: Approximately 101 sentinel general practitioners operatingMay-September	An acute respiratory tract infection with abrupt onset of at least two of the following: fever, chills, headache and myalgia
	2009: Approximately 101 sentinel general practitioners operating May-December (due to pandemic)	As above
Philippines	59 health centres and hospitals	Fever of >38°C and cough or sore throat. For children ≤3 years, fever of >38°C and cough,sore throat or runny nose
Republic of Korea	Approximately 800 sentinel sites	WHO definition∧
Singapore	18 government clinics and 98 general practitioner clinics	WHO definition∧
Viet Nam:		
Hanoi	2006–2010: 15 sentinel hospitals	WHO definition∧
Ho Chi Minh City	2006: 3 sentinel hospitals	WHO definition∧
	2007–2010: 5 sentinel hospitals	As above

* Laboratory-confirmed cases of influenza are nationally notifiable.

∧ WHO definition: A person with sudden onset of fever of >38°C and cough or sore throat in the absence of other diagnoses.

All 14 countries reported using a sentinel site approach for influenza surveillance (Table 1). The number and type of sites varied widely across countries and areas, and some countries incrementally increased the number of surveillance sites during 2006–2010, especially in response to the A(H1N1)pdm09 pandemic. Confirmatory testing of a proportion of ILI cases was conducted through the surveillance systems. The number of specimens collected by surveillance schemes varied within and across countries and areas. Data were not collected on methods for selecting cases for specimen collection.

**Figure 2:**
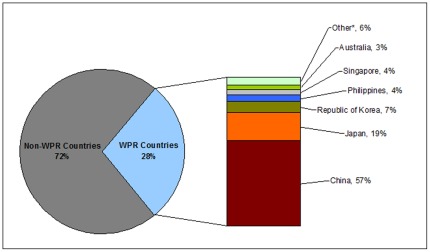
**Proportion of influenza specimens reported by Western Pacific Region countries to GISN, 2006–2010** ^∧^ **.** ^∧^ The proportion of contribution of viruses reported to FluNet from NICs in the Western Pacific Region ranged from 25–43% during 2006–2010. * Other: Viet Nam, Mongolia, New Zealand, Cambodia, Malaysia, New Caledonia (France), Fiji and Lao PDR.

During the study period, 1,070,792 influenza virus detections were reported to FluNet globally, of which 301,195 (28%) were reported from NICs in the Western Pacific Region (Figure 2). Within the Western Pacific Region, China contributed the most data for virus detections (57%), followed by Japan (19%) and the Republic of Korea (7%).

The number of Western Pacific Region countries submitting virus information to FluNet and the reported number of specimens tested for influenza increased over the five-year study period (Table 2). From 2006 through 2008, an average of 84,105 specimens was tested each year (range: 65,103 to 94,274). In 2009, the number of specimens tested increased to 366,164, a 4.3-fold compared to the average tested in the previous three years, and remained elevated in 2010. Of the total 926,064 specimens tested throughout the study period, 21.2% (n = 196,720) were influenza positive. From 2006 through 2008, the weighted average proportion of specimens found positive for influenza was 11.7% (range: 11.4% to 12.0%). The overall proportion of specimens found positive for influenza increased 2.7-fold to 31.6% in 2009 compared to the 2006–2008 average, but decreased to 16.8% in 2010.

**Table 2:** Specimens tested and specimens positive for influenza by type/subtype/lineage in Western Pacific Region countries, 2006–2010. ^∧^

	2006°		2007^1^	2008^2^	2009^3^	2010^3^
Number of specimens tested	**65,103**		**92,939**	**94,274**	**366,164**	**307,584**
Number of influenza positive specimens	**7,425 (11.4%)**		**11,143 (12.0%)**	**11,025 (11.7%)**	**115,554 (31.6%)**	**51,573 (16.8%)**
Influenza positive specimens by type/subtype						
Influenza A total	***4,393***		***7,297***	***7,426***	***110,668***	***26,008***
A(H1)	2,952	907		4,241	6,307	31
A(H1N1)pdm09	0	0		0	74,252	10,728
A(H3)	918	5,397		1,961	19,018	12,276
A(subtyping not performed)	523	993		1,224	11,091	2,973
Influenza B total	***3,032***		***3,846***	***3,599***	***4,886***	***25,565***
B(Victoria)	744	927		827	1,532	4,505
B(Yamagata)	76	1,642		1,360	235	954
B(lineage not determined)	2,212	1,277		1,412	3,119	20,106

°Data from Australia, Cambodia, China, Malaysia, Mongolia, New Caledonia (France), New Zealand, Philippines, and Viet Nam.

^1^Data from the countries with data in 2006 plus Singapore.

^2^Data from the countries with data in 2007 plus the Republic of Korea and Lao PDR.

^3^Data from the countries with data in 2008 plus Fiji.

Influenza A viruses accounted for the majority of viruses reported between 2006 and 2009, but an equal proportion of influenza A and influenza B viruses was detected in 2010. By subtype or lineage, a large proportion of viruses reported were seasonal A(H1) (40%) and B (lineage not determined) (30%) in 2006. This changed in 2007 when 48% of all influenza positive specimens were A(H3), which continued to be commonly detected in 2008 (18%) along with a resurgence of seasonal A(H1) viruses (38%).

In 2009, 64% of all viruses reported were A(H1N1)pdm09. Other viruses detected in 2009 were A(H3) (16%) and A(not subtyped) (10%), but very few influenza B viruses were reported. However, there was an increase in influenza B(lineage not determined) reports in 2010 (39% of all viruses reported) along with frequent detection of the A(H1N1)pdm09 and A(H3). B(Victoria) was reported more frequently than B(Yamagata) in 2006, 2009 and 2010 but less frequently in 2007 and 2008.

ILI case numbers and total all-cause consultations were provided by 10 of the 14 countries in this study. Australia, China (including Hong Kong), the Republic of Korea, Malaysia, Mongolia and Viet Nam provided data for the five year study period. New Zealand provided aggregate data for the five years, and was analyzed separately. Japan provided the total number of ILI cases but not the total number of all-cause consultations for the study period, and was also analyzed separately. Lao PDR provided ILI data for three years (2008–2010) and Cambodia, the Philippines and Singapore provided ILI data for two years (2009–2010) (Figure 1). Data for total specimens tested and number positive for influenza were available for all countries except Japan, where only the number of specimens positive for influenza was provided.

**Figure 3:**
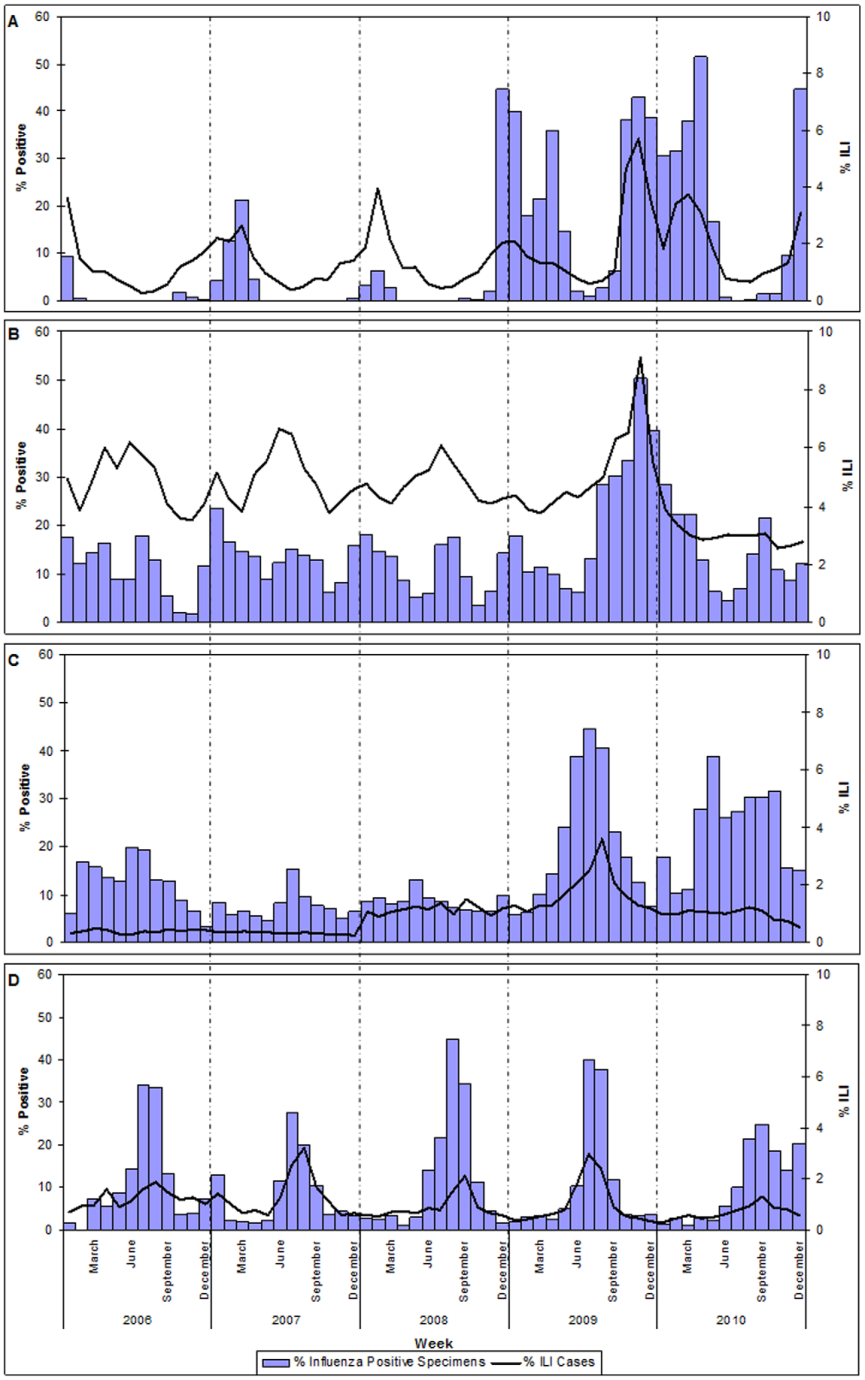
Proportion of specimens positive for influenza and proportion of consultations meeting ILI case definition in Western Pacific Region countries, 2006–2010.* ^∧^ *Panel A: Mongolia and Republic of Korea; Panel B: China; Panel C: Cambodia, Lao PDR, Malaysia, Philippines, Singapore, and Viet Nam; and Panel D: Australia, Fiji, New Caledonia (France), and New Zealand. ^∧^ Panel D includes proportion of specimens positive for influenza for all four countries, but ILI ratios only for Australia.

Over the five year period, peaks in ILI reporting appeared to be coincident with the proportion of specimens positive for influenza (Figure 3). This was particularly evident for the northern and southern hemispheres (Panels A & D, Figure 3).

A yearly seasonal pattern typical of temperate northern hemisphere countries was observed in the Republic of Korea and Mongolia (Panel A, Figure 3), where peaks in ILI activity were detected in January-March each year. A similar seasonal pattern was observed in Japan, where the reported number of ILI cases and the number of specimens positive for influenza were greatest in the first three months of each year (data not shown). A seasonal pattern typical of temperate southern hemisphere countries was observed in Australia (Panel D, Figure 3), where ILI activity and specimens positive for influenza peaked in July-September each year. A similar pattern was observed in New Zealand where ILI activity peaked approximately five weeks earlier than it did in Australia each year (data not shown). Two annual peaks in activity were observed in China (Panel B, Figure 3), one in January and one in July or August, from 2006 through 2008 and prior to the appearance of influenza A(H1N1)pdm09 in 2009. A temporal pattern was less evident in the group of six other reporting countries (Panel C, Figure 3).

ILI activity and the proportion of specimens positive for influenza were greater during July through December 2009 compared to previous years. This was observed in all reporting countries except Australia, where peak ILI activity in 2009 was of similar magnitude to previous years.

**Figure 4:**
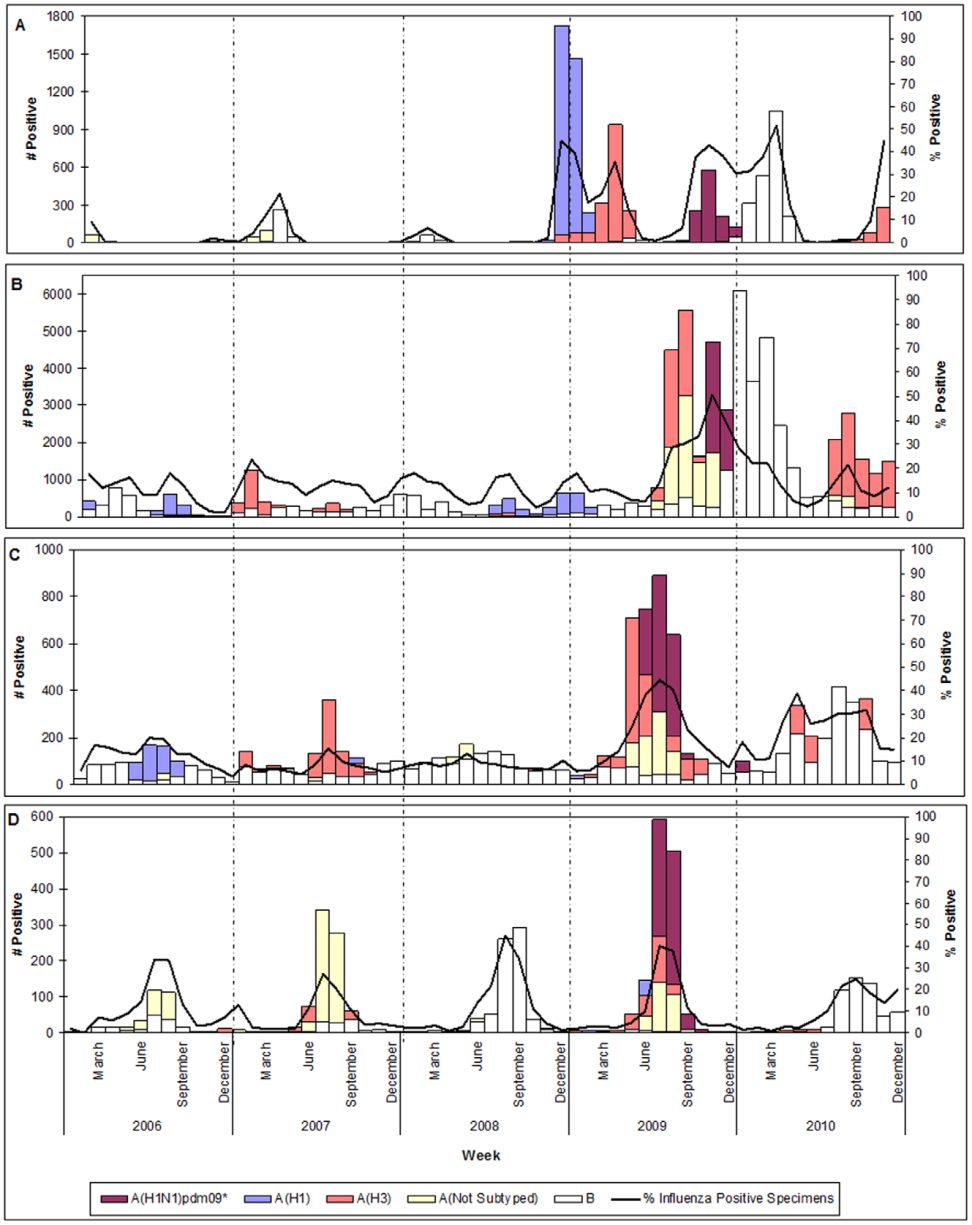
Number of influenza viruses by type/subtype and proportion of specimens positive for influenza in Western Pacific Region countries, 2006–2010.* ^∧^ * Panel A: Mongolia and Republic of Korea; Panel B: China; Panel C: Cambodia, Lao PDR, Malaysia, Philippines, Singapore, Viet Nam; and Panel D: Australia, Fiji, New Caledonia (France), New Zealand. ^∧^ The number of reported A(H1N1)pdm09 viruses was very high compared to other subtypes/lineages. Thus, to better illustrate the patterns for other subtypes/lineages, the A(H1N1)pdm09 numbers were divided by five.

In 2006 and 2007, the predominant influenza viruses in the region were influenza B and seasonal A(H1) and influenza B and influenza A(H3), respectively. Influenza B was identified throughout this period, seasonal A(H1) was frequently identified in May through August 2006 and A(H3) detection increased in January through August 2007. This was followed by detection of A(H3) in Australia, Fiji, New Zealand and New Caledonia (France) in June through September 2007 (Panel D, Figure 4). Only influenza B and A(not subtyped) were detected in Mongolia and the Republic of Korea during the same time period.

In 2008, influenza B was most frequently reported by countries in the region. However, in the latter half of 2008 an increase in seasonal A(H1) was seen in China (Panel B, Figure 4), Japan (data not shown), Mongolia and the Republic of Korea (Panel A, Figure 4). Seasonal A(H1) viruses were reported from Mongolia from December 2008 through February 2009, but did not predominate among influenza viruses reported by other countries in the Western Pacific Region during 2008 (Panels C and D, Figure 4).

In 2009, pandemic A(H1N1)pdm09 was predominant in the region. A(H3) viruses were reported from all countries in the region, and peaked earliest in Japan, Mongolia and the Republic of Korea, followed by all other countries except China which saw a later A(H3) peak in August through September. Australia, the Philippines and Singapore were the first countries to report detection of the pandemic A(H1N1)pdm09 subtype to FluNet in May 2009 (although New Zealand was the first country in the region to detect this virus). The dominance of this virus in the Western Pacific Region countries was staggered, peaking during May to August 2009 in Cambodia, Lao PDR, Malaysia, Philippines, Singapore and Viet Nam, July to August 2009 in Australia, Fiji, New Zealand and New Caledonia (France), and October 2009 to January 2010 in China, Mongolia and the Republic of Korea. Japan had dual peaks of pandemic virus detection, with a large number of detections in August to September 2009 followed by resurgence in November to January 2010 (data not shown).

During the pandemic period (11 June 2009 to 10 August 2010), other influenza A virus sub-types, including A(H3), were reported. There was also resurgence of influenza B activity with peaks in China, Mongolia and the Republic of Korea during January to April 2010, followed by the remaining countries in July to September 2010 (Figure 4).

Six countries reported data by age group for ILI cases and total consultations: Cambodia (2009–2010), Lao PDR (2008–2010), Mongolia (2006–2010), New Zealand (2006–2010), the Philippines (2009–2010) and Viet Nam (2008–2010). Cambodia, Mongolia and New Zealand reported greater ILI activity in the 0–5 years age group than other age groups (5 to <18, 18 to <65, and 65 years and over, data not shown). The Philippines and Lao PDR reported greater ILI activity in the 0 to <5 and 5 to <18 age groups, while Viet Nam reported greater ILI activity in the 5 to <18 and 18 to <65 age groups (data not shown).

## Discussion

Influenza was prevalent in all countries and areas that participated in this study, which included countries and areas in temperate parts of the northern and southern hemispheres and countries and areas in the tropics. Despite differences in surveillance approaches, methods and intensity, commonalities in disease and virus circulation patterns were identified.

The seasonal pattern of influenza activity observed in Mongolia and the Republic of Korea in the northern hemisphere is consistent with a previous report from Mongolia [7]. Similarities in seasonality were also observed amongst Australia, Fiji, New Caledonia (France) and New Zealand in the southern hemisphere. Other countries in the region had less discernable seasonal patterns with influenza activity occurring throughout the year, which is aligned with previous research findings and generally consistent with their location in the tropics [6], [8], [9], . Although Fiji and New Caledonia (France) are tropical, their influenza circulation patterns appeared to be more aligned with Australia and New Zealand than other tropical countries in the region. This highlights that influenza circulation may be dependent on a multitude of factors including seasonality and people movement pathways. Patterns of disease may also vary within countries, such as China where research suggests that seasonal patterns and peaks of influenza activity differ between the northern and the southern parts of the country [9].

Detection of A(H3) in Australia and New Zealand in mid-2007 was preceded by frequent detection of the virus in Cambodia, China, Lao PDR, Malaysia, the Philippines, Singapore and Viet Nam in early 2007. This supports the hypothesis that dominant strains in East and Southeast Asian countries spread to other countries in the region [11], [12], [13], [14]. Similarly, the dominance of former seasonal A(H1) in Mongolia and the Republic of Korea in December 2008 to February 2009 was preceded by dominance of this subtype in China in mid-2008. In the future, timely reporting and regional sharing of influenza virus information may serve as an early warning, and may assist countries to anticipate the potential severity and burden associated with incoming influenza strains. One example of this in practice is in the Federated States of Micronesia which has utilized the influenza surveillance findings from Guam for early warning (personal communication). Efforts are ongoing within GISRS in the region to increase timely reporting and regional sharing of influenza virus information to maximize the utility of these surveillance systems.

This study included data on the influenza A(H1N1)pdm09 pandemic, providing the opportunity to assess changes in patterns of influenza activity during and after this outlier event. The novel A(H1N1)pdm09 virus was first detected in Mexico in April 2009. Despite rapid spread to the Western Pacific Region, pandemic virus activity peaked in the temperate countries during their typical annual influenza seasons: the winter months in Australia and New Zealand (July to September) and in Japan, Mongolia and the Republic of Korea (November to January). Even though this virus dominated epidemiological patterns in 2009 to 2010, other types/subtypes of influenza co-circulated at low levels during the pandemic period throughout the region [15]. The epidemiological characteristics of the pandemic in the region have been described previously [5].

Overall, temporal patterns of influenza activity appeared to be coincident between ILI syndromic surveillance and virological surveillance. This highlights the utility of combining data from ILI surveillance with virological surveillance for interpreting influenza activity. Virological surveillance adds specificity to the overall influenza surveillance program, informs vaccine strain selection at the global level and enables prompt detection of antigenic, genomic and drug-sensitivity changes in the virus. An example of the latter was the detection of increased antiviral drug resistance among seasonal A(H1) strains in 2008 [16]. This highlights the importance of timely reporting into these surveillance systems, including FluNet, so that virus detection is synchronized with disease activity and controls measures.

This study highlighted that regional participation in global virological surveillance systems has been high. Of the 34 different virus strains recommended by WHO for use in influenza vaccines between 1988 and 2010, 26 (76.4%) were developed from isolates from this region [17]. Participation increased during the study period as evidenced by the addition of three NICs, one each in Cambodia, Lao PDR and Viet Nam, and one WHO Collaborating Centre in China. There was also an increase in the number of sentinel surveillance sites in some countries.

This study had a number of limitations. A key limitation of the study is that the data are aggregated across countries, over time and age groups. This limits the type of analyses and inferences made about the data. Since data aggregation can result in reporting bias, we have tried to reduce the risk of bias through the involvement of authors from all countries participating in the study. Authors from each country were tasked with ensuring that the analyses and interpretation were in line with their national-level context and conclusions.

Reporting countries and areas applied different case definitions, as well as different methodologies for data collection and sampling, and some incrementally added surveillance sites or changed surveillance methods or testing methods. These differences limited the ability to make comparisons and draw conclusions, especially for the magnitude of disease activity and representativeness of the surveillance systems.

Use of different ILI case definitions impacts disease prevalence comparisons, where some definitions may be more sensitive than others in case-ascertainment. Even though the ILI case definitions used by the 14 reporting countries appear relatively similar, the impact of the differences on the magnitude of disease activity was not assessed. This is an area of future work. It may be argued that consistent use of different case definitions over time still enable comparison of disease trends. However, in this study, the data collection tool did not assess whether countries changed their ILI case definitions during the five year period (2006–2010). Again, this necessitates further research to determine whether case definitions have changed over time and the impact changes may have had on reported ILI trends.

Although the data collection tools included disaggregation by age groupings, the available data was too limited to do summary analyses. In addition, not all influenza surveillance information in the region was captured. For example, countries and areas without NICs in the Pacific also contribute virus data to the international influenza surveillance systems by submitting samples to reference laboratories for testing, but their data were not included in this study.

### Conclusions and Way Forward

This collaborative effort to summarize epidemiological and virological characteristics of influenza adds to the body of knowledge on influenza in the Western Pacific Region. This study highlights the progress made in influenza surveillance capacity in countries of the Western Pacific Region between 2006 and 2010. Epidemiological and virological characteristics of influenza appeared to be interrelated across countries, underscoring the importance of information-sharing and collaboration. However, it is clear that variations in data collection, case definitions and testing procedures currently limit comparisons.

Regional strategies have been developed to further strengthen influenza surveillance and research in the region and address the current limitations in cross-region analysis [18]. Countries and areas are encouraged to develop detailed workplans and monitor progress in three areas of work: (1) defining the epidemiology and burden of influenza; (2) improving virological testing capacity; and (3) improving communication and reporting through the development or strengthening of regional and global networks.

Through implementation of workplans to strengthen influenza surveillance, conduct of the research agenda and regular information-sharing about epidemiological findings, policy makers in the Western Pacific Region will be able to refine their policies and enhance influenza control based on a solid foundation of knowledge of the burden and characteristics of influenza virus infection in their own and neighbouring countries.

## Supporting Information

Table S1 **Type and period of available data for this study from the participating Western Pacific Region countries.** *These data were used as denominators to calculate proportions of ILI and influenza positive specimens, respectively. ^∧^ Data on total consultations and total specimens tested were not provided, therefore data were analyzed separately. # Data on ILI cases and total consultations were provided aggregated as rates, therefore data were analyzed separately.
(DOC)
