# Supplementary material for: Epidemiological and Virological Characteristics of Influenza in the Western Pacific Region of the World Health Organization, 2006–2010
Source: PLoS One. 2012 May 29;7(5):e37568. doi: 10.1371/journal.pone.0037568 (PMC3366627; doi:10.1371/journal.pone.0037568)
Supplement: Table S1 — Type and period of available data for this study from the participating Western Pacific Region countries. *These data were used as denominators to calculate proportions of ILI and influenza positive specimens, respectively. ∧ Data on total consultations and total specimens tested were not provided, therefore data were analyzed separately. # Data on ILI cases and total consultations were provided aggregated as rates, therefore data were analyzed separately. (DOC) [file pone.0037568.s001.doc]

| Country | ILI cases | Total consultations* | Age data for ILI cases and total consultations | Influenza positive specimens | Total specimens tested* |
| --- | --- | --- | --- | --- | --- |
| Australia | 2006-2010 | 2006-2010 | N/A | 2006-2010 | 2006-2010 |
| Cambodia | 2009-2010 | 2009-2010 | 2009-2010 | 2006-2010 | 2006-2010 |
| China | 2006-2010 | 2006-2010 | N/A | 2006-2010 | 2006-2010 |
| Fiji | N/A | N/A | N/A | 2009-2010 | 2009-2010 |
| Japan^ | 2006-2010 | N/A | N/A | 2006-2010 | N/A |
| Lao PDR | 2008-2010 | 2008-2010 | 2008-2010 | 2008-2010 | 2008-2010 |
| Malaysia | 2006-2010 | 2006-2010 | N/A | 2006-2010 | 2006-2010 |
| Mongolia | 2006-2010 | 2006-2010 | 2006-2010 | 2006-2010 | 2006-2010 |
| New Caledonia (France) | N/A | N/A | N/A | 2006-2010 | 2006-2010 |
| New Zealand# | 2006-2010 | 2006-2010 | 2006-2010 | 2006-2010 | 2006-2010 |
| Philippines | 2009-2010 | 2009-2010 | 2009-2010 | 2006-2010 | 2006-2010 |
| Republic of Korea | 2006-2010 | 2006-2010 | N/A | 2008-2010 | 2008-2010 |
| Singapore | 2009-2010 | 2009-2010 | N/A | 2007-2010 | 2007-2010 |
| Viet Nam | 2006-2010 | 2006-2010 | 2008-2010 | 2006-2010 | 2006-2010 |
